# A microRNA signature of toxic extrasynaptic *N*-methyl-D-aspartate (NMDA) receptor signaling

**DOI:** 10.1186/s13041-020-0546-0

**Published:** 2020-01-10

**Authors:** Carlos Bas-Orth, Mirja Koch, David Lau, Bettina Buchthal, Hilmar Bading

**Affiliations:** 1 Department of Neurobiology, Interdisciplinary Center for Neurosciences, Heidelberg University, 69120 Heidelberg, Germany; 2 Department of Medical Cell Biology, Institute for Anatomy and Cell Biology, Heidelberg University, Im Neuenheimer Feld 307, 69120 Heidelberg, Germany

**Keywords:** MicroRNA, NMDA receptor, Neurotransmitter receptors, Cell death, Neurodegeneration, Kainate, Seizures, Biomarkers

## Abstract

The cellular consequences of N-Methyl-D-Aspartate receptor (NMDAR) stimulation depend on the receptors’ subcellular localization. Synaptic NMDARs promote plasticity and survival whereas extrasynaptic NMDARs mediate excitotoxicity and contribute to cell death in neurodegenerative diseases. The mechanisms that couple activation of extrasynaptic NMDARs to cell death remain incompletely understood. We here show that activation of extrasynaptic NMDARs by bath application of NMDA or L-glutamate leads to the upregulation of a group of 19 microRNAs in cultured mouse hippocampal neurons. In contrast, none of these microRNAs is induced upon stimulation of synaptic activity. Increased microRNA expression depends on the pri-miRNA processing enzyme Drosha, but not on de novo gene transcription. These findings suggest that toxic NMDAR signaling involves changes in the expression levels of particular microRNAs.

## Introduction

MicroRNAs (miRNAs) are a class of small non-coding RNAs that act as post-transcriptional regulators of gene expression. They repress the expression of their target genes by inhibiting mRNA translation and/or by mediating mRNA degradation [1, 2]. miRNAs predominantly function in a dose-dependent manner to precisely adjust the expression levels of their target genes [2, 3]. Accordingly, miRNA expression itself needs to be tightly controlled and maintained at levels that meet cellular needs. In neurons, miRNA levels are subject to regulation by external cues. For example, sensory experience, synaptic activity, and glutamatergic signaling have been reported to induce specific miRNAs [4–12] which enable structural and functional plasticity by fine tuning the levels of plasticity-related genes [4, 13–15]. However, miRNAs are also upregulated under pathological conditions like ischemia, spinal cord injury, neurodegenerative diseases, and neuropsychiatric disorders [16–23]. Under these conditions miRNAs promote neuronal dysfunction and cell death. It remains unclear what distinguishes plasticity-associated from pathological miRNA regulation at the molecular level; however, both physiological and disease-associated miRNA regulation are thought to involve glutamatergic signaling. A key determinant that defines physiological vs. pathological outcomes of glutamatergic signaling is the activation of synaptic vs. extrasynaptic NMDA-receptors (NMDARs), respectively [24–26]. Synaptic NMDARs promote synaptic plasticity, learning, and neuronal survival while extrasynaptic NMDARs link to cell death pathways and disease [24, 27–29]. Here we investigated the possibility that differential regulation of miRNAs by toxic versus survival promoting NMDARs could potentially explain the differences in physiological vs. pathological miRNA expression. Using microArray and quantitative real-time PCR (qRT-PCR) analyses we identified a set of miRNAs that are specifically induced by toxic NMDAR signaling.

## Materials and methods

### Animals and ethics statement

C57BL/6NCrl mice (Charles River) and Crl:SD Sprague-Dawley rats (Charles River), were used in this study. Animals were maintained in pathogen-free and light- (12 h light/ 12 h dark) and temperature-controlled (22 °C ± 2 °C) conditions and had ad libitum access to water and food. Animals were group-housed in conventional cages and were provided with environmental enrichment. All procedures were done in accordance with German guidelines for the care and use of laboratory animals and with the European Community Council Directive 2010/63/EU. Experiments were approved by local authorities.

### Cell culture

Hippocampal neurons from newborn C57BL/6 mice were prepared and maintained as described previously [30]. In brief, neurons were grown in Neurobasal-A medium (Life Technologies) supplemented with B27 (Life Technologies), 0.5 mM glutamine, and 1% rat serum. On day in vitro (DIV) 8 growth medium was exchanged with transfection medium [31] consisting of a mixture of buffered salt-glucose-glycine (SGG) solution [10 mM Hepes (pH 7.4), 114 mM NaCI, 26.1 mM NaHCO3, 5.3 mM KCI, 1 mM MgCI2, 2 mM CaCI2, 30 mM glucose, 1 mM glycine, 0.5 mM sodium pyruvate, and 0.001% phenol red] and phosphate free Eagle’s minimum essential medium (MEM) (9:1 vol:vol), supplemented with insulin (7.5 μg/ml), transferrin (7.5 μg/mI), sodium selenite (7.5 ng/ml) (ITS supplement, Sigma-Aldrich). Experiments were performed after a culturing period of 10–12 DIV during which hippocampal neurons express functional glutamate receptors and develop an extensive network of synaptic contacts.

### Drug treatment

The following drugs were used in this study: N-Methyl-D-aspartic acid (NMDA, Sigma-Aldrich, 20-30 μM), bicuculline (Axxora, 50 μM), 4-aminopyridine (4-AP, Sigma-Aldrich, 2 mM), recombinant human BDNF (Peprotech, 100 ng/ml), glutamate (Sigma-Aldrich, 30 μM), MK-801 (Tocris, 10 μM), actinomycin D (Applichem, 10 μg/ml), α-Amanitin (Merck, 10 μg/ml). NMDA was added to cells at a final concentration of 20-30 μM. Cells were then placed in the incubator for 10 min, washed three times with fresh medium and returned to the incubator for the indicated times. Inhibitors were added 15–30 min before NMDA treatment and were included in all subsequent wash steps. KCl stimulation was performed by adding 0.41 volumes of depolarization solution containing 170 mM KCl, 2 mM CaCl2, 1 mM MgCl2, 10 mM HEPES.

### Induction of acute excitotoxic seizures

Six weeks old Sprague Dawley rats were administered with kainic acid (KA, Biotrend, 10 mg/kg i.p., dissolved in 0.9% saline, *n* = 14) or vehicle (phosphate-buffered saline, PBS, n = 14), to induce epileptic seizures. A trained observer monitored the severity of epileptic seizures for 4 h to categorize according to following criteria: level 1, immobility; level 2, forelimb and tail extension, rigid posture; level 3, repetitive movements, head bobbing; level 4, rearing and falling; level 5, continuous rearing and falling; level 6, severe tonic-clonic seizure; level 7, death [32]. Only animals that exhibited level 4 to 6 of epileptic seizure behavior were included in the analysis. 4 h after administration of KA or vehicle animals were killed by cervical dislocation. Brains were removed quickly and hippocampi were dissected in ice-cold dissection medium [30] containing 1 mM kynurenic acid (Sigma) and 10 mM MgCl_2_. Individual hippocampi were homogenized in 700 μl of Qiazol reagent (Qiagen) and total RNA was isolated as described below.

### RNA isolation

Total RNA was isolated using the miRNeasy kit (Qiagen) according to the manufacturer’s instructions.

### MicroRNA microarrays

For each condition (control, NMDA-treated, bicuculline-treated) three replicate samples from independent cell preparations were analyzed using mouse microRNA Microarrays (Agilent Technologies, Release 12.0) that profile 627 mouse miRNAs. Microarray analysis was performed at the genomics core facility of the German Cancer Research Center (DKFZ, Heidelberg, Germany). *P*-values were determined by student’s *t* test and Benjamini-Hochberg correction. To identify microRNAs that are increased by NMDA or bicuculline we chose a 20% change in expression as lower cut-off. This threshold was chosen because, first, previously reported stimulus-induced changes in neuronal miRNA expression are mostly rather low and, second, fold-changes are usually compressed in microArray analyses as compared to qRT-PCR.

### Quantitative real-time PCR

For analysis of miRNA expression, 10 ng of total RNA were reverse transcribed in a total volume of 15 μl using the High Capacity cDNA Reverse Transcription kit and miRNA-specific RT primers (Applied Biosystems). PCR reactions were performed using the TaqMan MicroRNA Assay kit (Applied Biosystems). Each PCR reaction contained 1.33 μl of the RT reaction product, 10 μl of TaqMan 2x Universal PCR Master Mix, and 1 μl of 20x TaqMan MicroRNA Assay reagent in a total volume of 20 μl. Expression of miRNAs was normalized to endogenous snoRNA 202 (assay ID 001232) and/or rat snoRNA (assay ID 001718) expression for each sample using the ∆∆Ct method.

### Molecular biology and preparation of recombinant adeno-associated viruses (rAAV)

For the expression of shRNA, a rAAV vector was used that contains the U6 promoter for shRNA expression and a CaMKII promoter driving mCherry expression [33]. The following shRNA sequences were used (5′-3′): *drosha*: CAACAGTCATAGAATATGA [34], non-targeting control-shRNA: GTGCCAAGACGGGTAGTCA [35]. All rAAV vectors were generated by standard molecular biology techniques and verified by sequencing. Viral particles were produced and purified as described previously [36]. Neurons were infected with 2-5 × 10^9^ particles/ml on DIV 4–6, yielding a typical infection rate of 80–90% [33, 35, 36].

### Antibodies

Rabbit monoclonal anti-Drosha (1:1000; Cell Signaling #3364), mouse monoclonal anti-Tubulin (1:400,000; Sigma #T9026).

### Cell death assay

20 h after KCl treatment, cells were fixed with pre-warmed 4% paraformaldehyde for 15 min, washed with PBS and counterstained with Hoechst 33258 (1 μg/ml) for 10 min. Cells were mounted in mowiol and examined by fluorescence microscopy. Dead neurons were identified by amorphous or shrunken nuclei visualized with Hoechst as described previously [24, 37].

## Results

### Toxic NMDA receptor signaling regulates a select group of miRNAs

To identify miRNAs that are regulated by the survival promoting versus death inducing activities of NMDARs we compared the miRNA expression profiles of primary mouse hippocampal neurons that had been treated with either bicuculline (50 μM) or NMDA (30 μM) in the presence of the NMDAR co-agonist glycine (900 μM). Application of the GABA-A receptor antagonist, bicuculline, reduces tonic GABAergic inhibition of the neuronal network causing action potential (AP) bursting and stimulation of synaptic α-amino-3-hydroxy-5-methyl-4-isoxazolepropionic acid (AMPA) and NMDA receptors [24, 36, 38, 39]. In contrast to this synaptic stimulation protocol, NMDA bath application leads to the activation of both synaptic and extrasynaptic NMDARs. Signaling through extrasynaptic NMDARs is dominant over synaptic signaling and is toxic to neurons in vitro and in vivo [24, 25, 27, 28, 36, 40]. Using microRNA microArrays we screened for miRNAs that were induced by bath application of NMDA but not by bicuculline treatment. We detected an increased expression of two miRNAs, miR-132 and miR-212, 4 h after exposing the neurons to bicuculline (Table 1), which is in line with the well documented activity-dependent regulation of these miRNAs [8–12]. In contrast, 4 h after NMDA treatment the levels of 19 miRNAs were increased more than 20% (Table 1, Additional file 1: Table S1; complete microarray data available at GEO GSE47601).

**Table 1:** List of differentially expressed miRNAs detected by miRNA microArray

miRBase accession	miRNA name	NMDA		Bicuculline	
		fold change	p adj.	fold change	p adj.
MI0004654	mmu-miR-689	5.24	0.07	0.91	1.00
MIMAT0004893	mmu-miR-574-5p	2.38	0.10	1.02	1.00
MIMAT0005837	mmu-miR-1187	2.21	0.09	1.03	1.00
MIMAT0003469	mmu-miR-690	2.12	0.10	1.00	1.00
MIMAT0003499	mmu-miR-709	1.99	0.23	0.90	1.00
MIMAT0007867	mmu-miR-1895	1.87	0.15	0.99	1.00
MIMAT0005846	mmu-miR-467f	1.79	0.14	0.98	1.00
MIMAT0003731	mmu-miR-671-5p	1.73	0.10	0.97	1.00
MIMAT0005834	mmu-miR-466i-3p	1.50	0.10	0.99	1.00
MIMAT0003182	mmu-miR-494-3p	1.35	0.15	1.00	1.00
MIMAT0003505	mmu-miR-714	1.34	0.22	1.01	1.00
MIMAT0007864	mmu-miR-1897-5p	1.30	0.36	1.01	1.00
MIMAT0007874	mmu-miR-1904	1.30	0.21	1.02	1.00
MIMAT0004882	mmu-miR-466f-3p	1.28	0.18	0.97	1.00
MIMAT0007878	mmu-miR-1894-3p	1.28	0.22	1.00	1.00
MIMAT0003495	mmu-miR-705	1.28	0.15	1.02	1.00
MIMAT0005839	mmu-miR-669f-3p	1.22	0.15	0.99	1.00
MIMAT0003502	mmu-miR-712-5p	1.22	0.15	1.02	1.00
MIMAT0004883	mmu-miR-466 g	1.21	0.23	0.99	1.00
MIMAT0017053	mmu-miR-212	1.02	0.95	1.86	0.72
MIMAT0016984	mmu-miR-132	1.06	0.85	1.38	1.00

Differential expression of miRNAs after activation of extrasynaptic vs. synaptic signaling. miRNA microarray data for the most strongly regulated miRNAs are shown. Extrasynaptic signaling was induced by bath application of NMDA (20 μM). Synaptic signaling was induced by application of the GABA-A receptor antagonist, bicuculline (50 μM) to reduce tonic network inhibition and thereby increase global synaptic activity. Fold-changes compared to untreated cells were calculated from three independent experiments. *P*-values were determined by two-tailed t test and adjusted with Benjamini-Hochberg correction for multiple testing

**Fig. 1:**
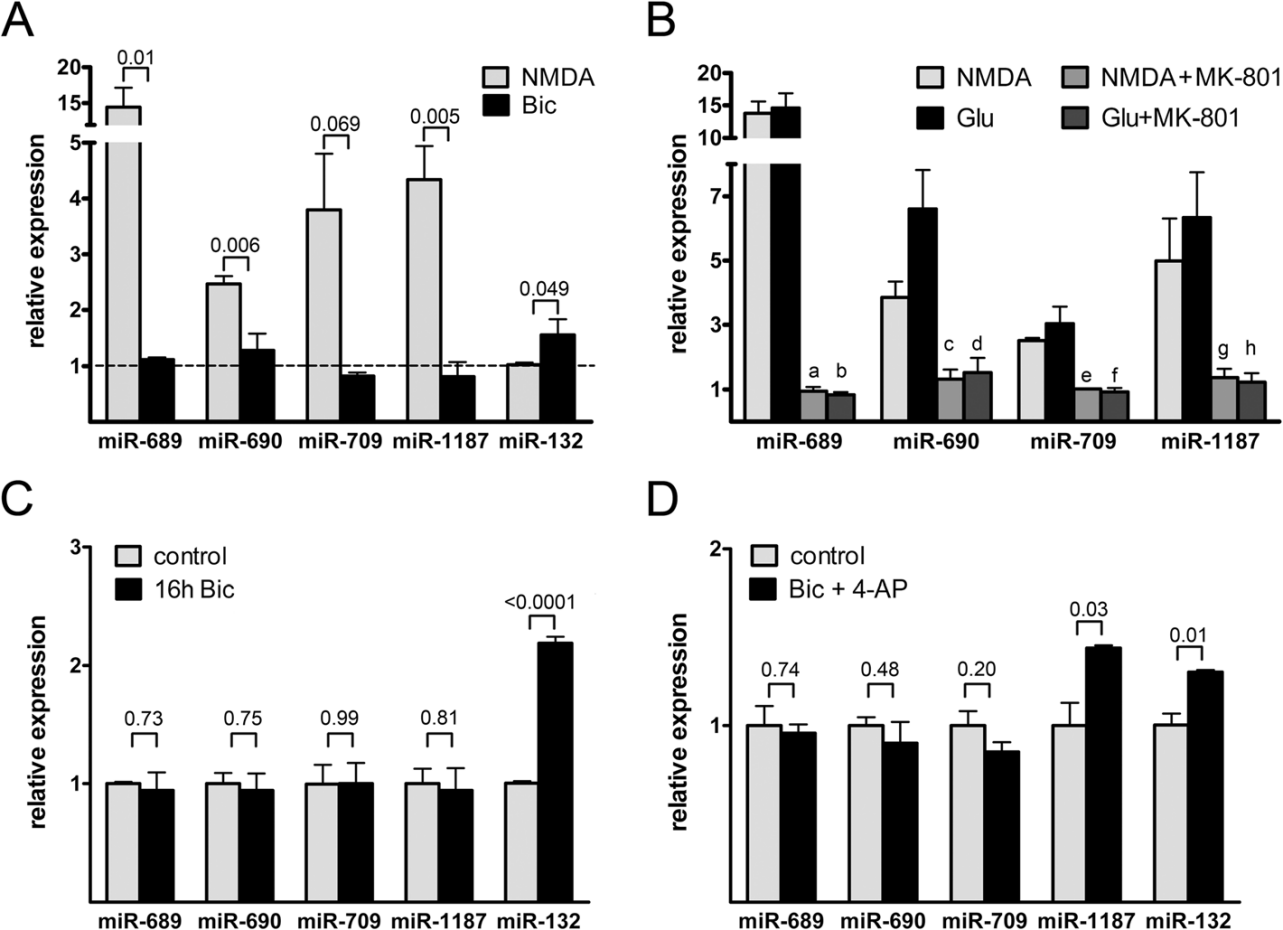
NMDA bath application increases the expression of several miRNAs. **a** QRT-PCR analysis of miRNA expression 4 h after stimulation with NMDA (30 μM) or bicuculline (Bic, 50 μM). All tested miRNAs except miR-132 are upregulated by NMDA but not by bicuculline. Conversely, miR-132 is upregulated by bicuculline but not by NMDA. Mean values (relative to untreated control) + SEM from five independent experiments are shown. **b** QRT-PCR analysis of miRNA expression 4 h after stimulation with NMDA (30 μM) or L-glutamate (Glu, 30 μM) in the presence or absence of MK-801 (10 μM). Mean values + SEM from three independent experiments are shown. **c** QRT-PCR analysis of miRNA expression 16 h after stimulation with bicuculline (Bic, 50 μM). None of the tested miRNAs, except miR-132, are upregulated by over-night treatment with bicuculline. Mean values + SEM from three independent experiments are shown. **d** QRT-PCR analysis of miRNA expression 4 h after stimulation with bicuculline (Bic, 50 μM) plus 4-aminopyridine (4-AP, 2 mM). Mean values + SEM from three independent experiments are shown. *P*-values were determined with two-tailed t test. P-values in *B* are NMDA vs NMDA + MK801: a = 0.002, c = 0.012, e < 0.0001, g = 0.055; Glu vs Glu + MK801: b = 0.004, d = 0.017, f = 0.018, h = 0.024

**Fig. 2:**
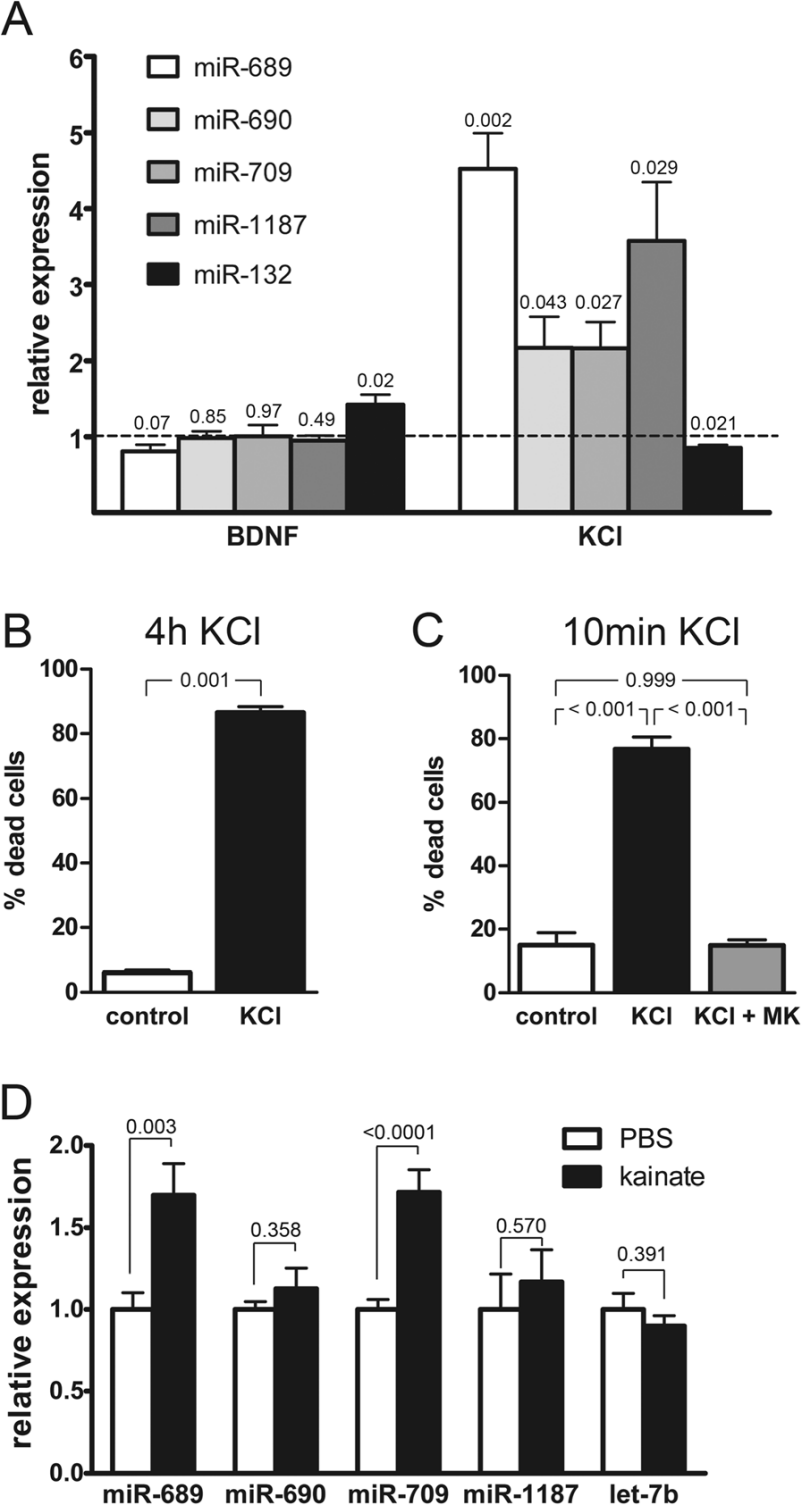
Differential expression of miRNAs by toxicity-associated vs. synaptic stimulation. **a** QRT-PCR analysis of miRNA expression 4 h after stimulation with indicated drugs. BDNF (brain derived neurotrophic factor, 100 ng/ml), KCl (50 mM potassium chloride). Mean values (relative to untreated control) + SEM from ≥3 independent experiments are shown. P-values were determined with two-tailed t test. **b*,*** **c** Analysis of cell death induced by 4 h (B) or 10 min (C) treatment of neurons with KCl (50 mM) in the presence or absence of the NMDAR antagonist, MK-801 (10 μM). Mean values + SEM from three independent experiments are shown. P-values were determined with two-tailed paired t test (**b**) and repeated-measures ANOVA with Tukey’s post test (**c**). **d** QRT-PCR analysis of miRNA expression in 6 week old rats 4 h after intraperitoneal injection of kainate (10 mg/kg) or vehicle (PBS). Mean values + SEM are shown. *N* = 14 animals per group from four independent experiments. P-values were determined with two-tailed t test

The two sets of miRNAs were non-overlapping, i.e., none of the NMDA-induced miRNAs were changed by bicuculline application and vice versa. The highest fold changes in induction were obtained for miR-689. However, it is currently under debate whether or not this small RNA is a true microRNA [41–43]. Nevertheless, due to its robust regulation by NMDA treatment, we selected this putative miRNA together with 3 additional miRNAs that were highly induced by NMDA for further analysis. We first verified the differential regulation of these miRNAs using RT-qPCR for mature miRNAs (Fig. 1a). We confirmed that NMDA treatment but also bath application of L-glutamate (30 μM) increased the levels of all 4 miRNAs (Fig. 1b). In the presence of the NMDAR antagonist, MK-801, glutamate had no effect on miRNA levels, demonstrating that NMDARs are necessary for miRNA induction. We next considered the possibility that extended episodes (> 4 h) of AP bursting could increase the levels of our group of miRNAs. However, RT-qPCR analysis revealed that also 16 h of bicuculline treatment did not induce changes in the levels of any of the miRNAs analyzed, except for the positive control miR-132 (Fig. 1c). These results are consistent with this set of miRNAs being specifically induced by the activation of extrasynaptic NMDARs. An alternative explanation for the different effects of NMDA vs. bicuculline might be the differently shaped Ca^2+^-signals (plateau vs. transients) evoked by these treatments. To test this we used a combined stimulation with bicuculline and the potassium channel blocker, 4-AP, which induces a sustained Ca^2+^ plateau comparable to NMDA bath application [24, 44]. Out of all miRNAs analyzed only miR-1187 and the positive control miR-132 were increased by this treatment (Fig. 1d). Thus for the majority of NMDA-induced miRNAs increased expression seems to depend on the site of Ca^2+^-entry rather than total Ca^2+^-load. To further rule out any regulation of these miRNAs by synaptic signaling we used two additional stimulation paradigms. First, we used bath application of BDNF (100 ng/ml). Similar to previous reports [4, 45, 46], 4 h BDNF application increased the levels of miR-132, but not those of miR-689, miR-690, miR-709, and miR-1187 (Fig. 2a). Second, we applied high extracellular concentrations of potassium (50 mM KCl), which results in sustained neuronal depolarization and calcium influx and is thus generally considered a model for electrical activation of neurons in vitro. We observed increased expression of miR-689, miR-690, miR-709, and miR-1187, but not of miR-132 after 4 h of KCl stimulation (Fig. 2a). This finding was unexpected and seemingly contradictory to the observed lack of regulation of expression of this group of miRNAs by synaptic activity (see Fig. 1). However, prolonged and strong depolarization of neurons might not adequately mimic synaptic stimulation, and could possibly impair cellular integrity. To investigate this, we analyzed cell viability 20 h after KCl treatment and found that both 10 min and 4 h of KCl stimulation resulted in severe cell death (Fig. 2b, c). KCl-induced cell death was completely blocked by the NMDAR antagonist, MK-801. These findings show that KCl treatment can cause severe glutamate-toxicity and warrant caution in the application and interpretation of this widely used stimulation paradigm. Together our results identify a set of miRNAs that are induced by toxicity-associated NMDAR signaling (i.e. induced by application of NMDA, glutamate, or KCl), but not by plasticity-associated synaptic signaling (i.e., induced by application of bicuculline or BDNF). We thus refer to these miRNAs as *t*oxicity-*a*ssociated *m*iRNAs, or TAMs.

### In vivo regulation of miRNAs

To investigate if TAM levels also increase under excitotoxic conditions in vivo, we induced epileptic seizures in young adult rats by intraperitoneal injection of kainic acid (KA) [32]. By activating KA receptors in hippocampal area CA3, KA triggers reverberating activity within the entire hippocampal formation which is propagated through NMDARs and triggers NMDAR-mediated excitotoxic cell death [47]. KA-induced brain damage can be attenuated by application of memantine [48], that at low dose preferentially blocks extrasynaptic NMDARs [49]. Four hours after KA injection the levels of two of the tested miRNAs, miR-689 and miR-709, were increased in the hippocampus (Fig. 2d). For two other miRNAs, miR-690 and miR-1187, we detected only a small increase in expression that was not statistically significant (miR-690, *p* = 0.36; miR-1187, *p* = 0.57). This may be due to a lack of sensitivity of the assay since in contrast to our pyramidal neuron-enriched primary cultures the hippocampal tissue in the in vivo experiment contains a mixed population of cell types and only in a subset of neurons expression of miRNAs may increase.

### NMDA-mediated increases in TAM levels are independent of transcription

**Fig. 3:**
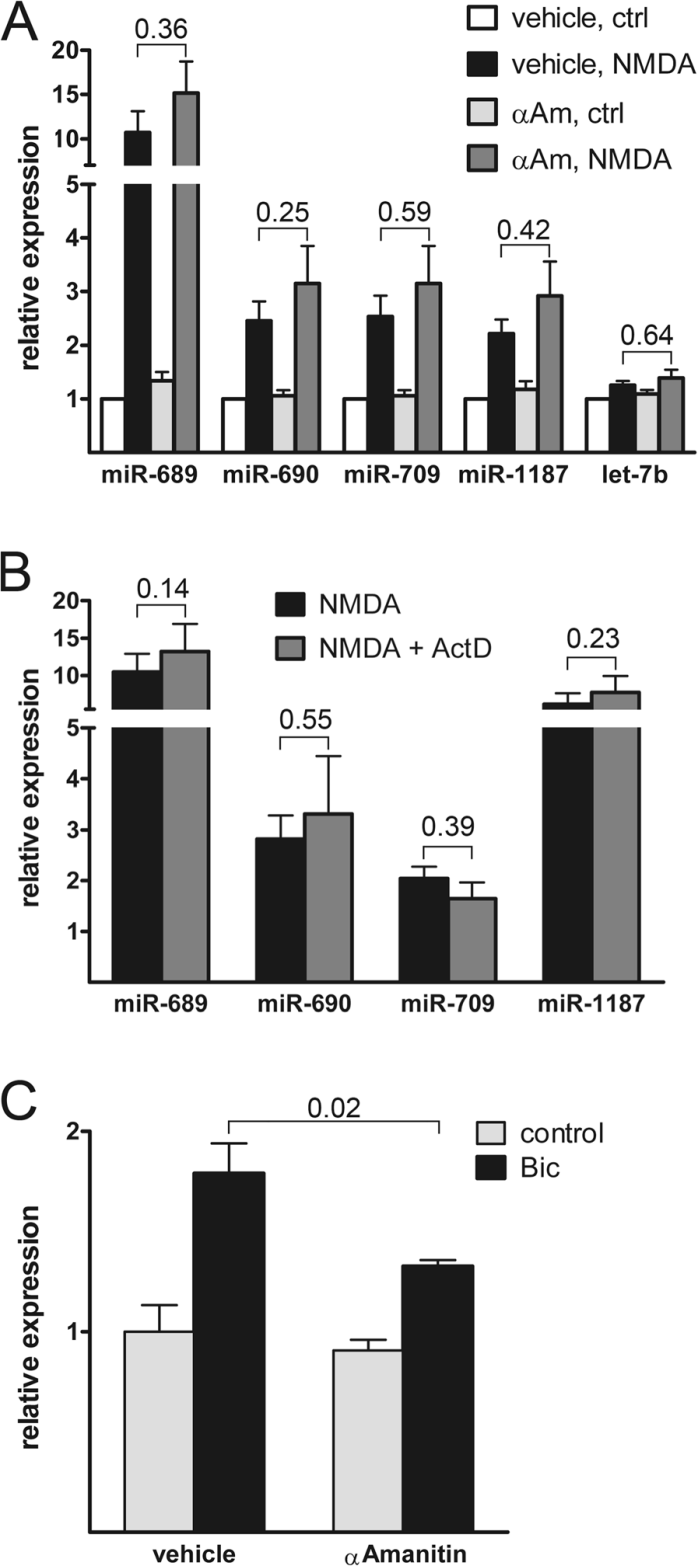
Transcription is not required for upregulation of toxicity-associated miRNAs. **a, b** QRT-PCR analysis of miRNA expression 4 h after stimulation with NMDA (30 μM) in the presence or absence of the transcription inhibitors alpha-Amanitin (**a**) or actinomycin D (**b**). Mean values + SEM from ≥3 independent experiments are shown. P-values were determined by repeated-measures ANOVA with Tukey’s post test (**a**) and two-tailed paired t test (**b**). **c** QRT-PCR analysis of miR-132 expression 4 h after stimulation with bicuculline (Bic, 50 μM) in the presence or absence of the transcription inhibitor alpha-Amanitin. Mean values + SEM from 4 independent experiments are shown. P-value was determined by two-tailed t test

Several studies on regulated expression of miRNAs have reported transcription-dependent mechanisms [4, 6, 12, 50, 51]. To investigate if, similarly, changes in TAM levels are mediated by increased transcription, we used two inhibitors of transcription, alpha-Amanitin and actinomycin D. In a control experiment alpha-Amanitin blocked the bicuculline-induced increase in miR-132 levels, demonstrating that we can detect transcription-dependent miRNA regulation with our assay (Fig. 3c). However, neither inhibitor blocked the NMDA-induced increase in TAM levels (Fig. 3a, b), indicating that under conditions of excitotoxicity TAMs are regulated at the post-transcriptional level.

### Drosha is required for NMDA-mediated TAM expression

**Fig. 4:**
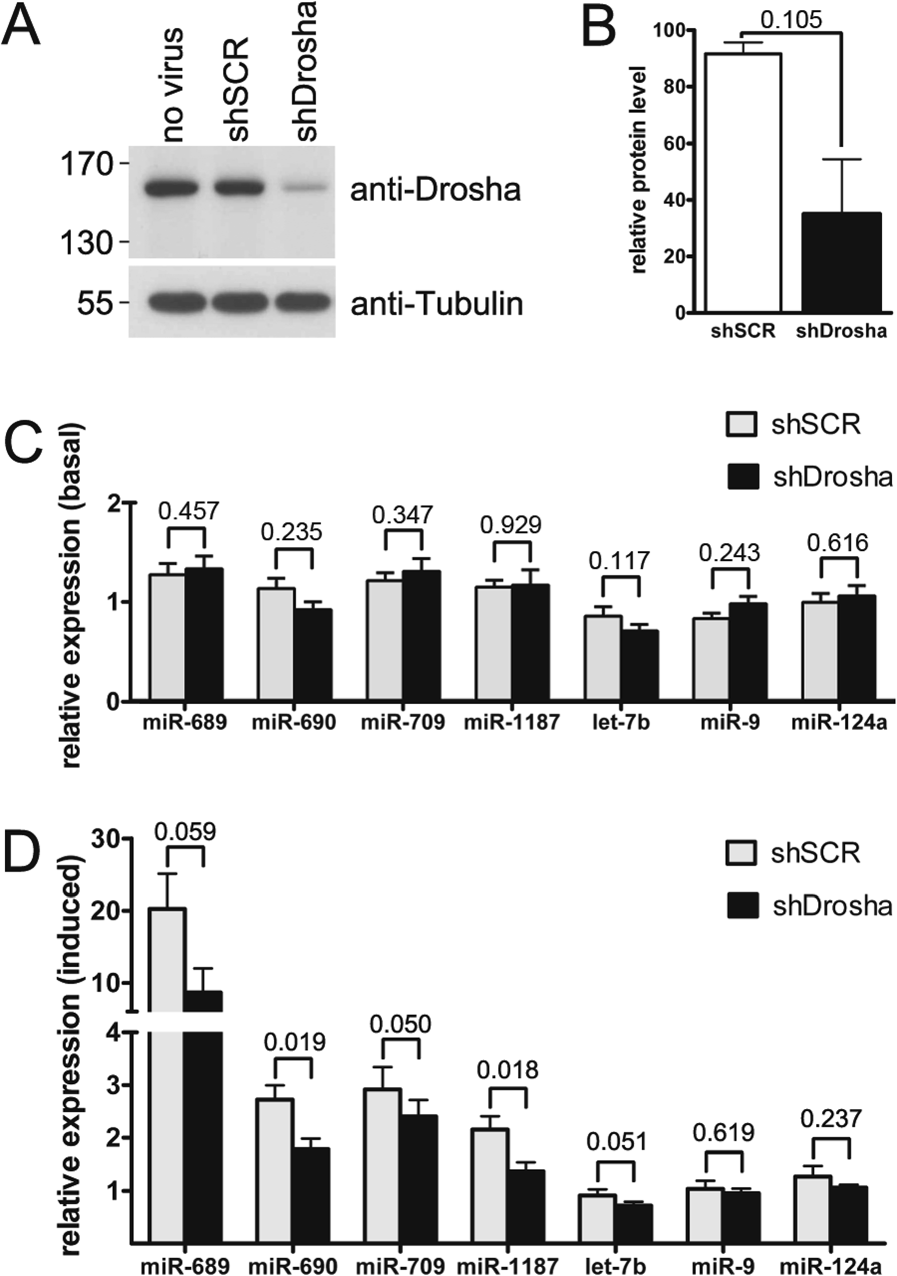
Drosha is required for NMDA-induced increases in TAM levels. **a** Representative Western Blot of hippocampal neurons that were left uninfected or that were infected with rAAV expressing scrambled or anti-Drosha shRNA. **b** Quantification of Western Blot experiments. DROSHA protein levels were calculated relative to uninfected controls. Mean + SEM from two independent experiments are shown. P-value was determined with two-tailed t test. Compared to uninfected and scrambled shRNA controls, targeting shRNA reduces DROSHA protein levels by approximately 60%. **c**, **d** QRT-PCR analysis of TAM and control miRNA expression in cells infected with rAAV expressing scrambled or anti-Drosha shRNA, without (basal, **c**) and with (induced, **d**) NMDA stimulation. Mean values (relative to uninfected control) + SEM from five independent experiments are shown. P-values were determined with two-tailed paired t test

Given the results from our transcription inhibition experiments we reasoned that NMDA-treatment might lead to elevated TAM levels via enhanced processing of pre-existing primary miRNA transcripts. To test this we knocked-down Drosha, a key enzyme of the miRNA biogenesis machinery [1], and measured basal and NMDA-induced expression levels of TAMs and a set of NMDA-independent control miRNAs (let-7b, miR-9, miR-124a). Infection of hippocampal neurons with rAAVs harboring a short-hairpin RNA that targets Drosha resulted in a ~ 60% reduction of DROSHA protein levels (Fig. 4a-b). This partial knock-down had no major effect on TAM or control miRNA levels under basal conditions (Fig. 4c, all changes less than 20%, statistically not significant), but it partially attenuated the NMDA-mediated increase in TAM expression (Fig. 4d, relative changes miR689: 57.2%, miR690: 34.5%, miR709: 17.6%, miR1187: 36.3%; miR690 and miR1187 statistically significant).

## Discussion

In this study we identified a group of miRNAs that increase in expression upon stimulation of the toxic but not of the survival promoting activity of NMDARs. This identifies the regulated expression of a subset of miRNAs as a novel component of cell death associated NMDAR signaling.

### miRNAs and neuropathology

In line with our findings, several previous studies have reported changes in miRNA expression under neuropathological conditions such as ischemic stroke, intracerebral hemorrhage, and epileptic seizures [20, 52–56]. While each of these studies identified up to several dozens of differentially regulated miRNAs, the overlap between individual studies (including our study) is very low. This is likely due to differences in animal models, experimental conditions and miRNA profiling methods used. Nevertheless, together these studies support a role for miRNAs in mediating diverse downstream effects of neuropathological insults.

### miRNAs and excitotoxicity

In this study, we focused on the divergent roles of synaptic versus extrasynaptic NMDAR signaling in the regulation of miRNA expression. Several lines of evidence support our conclusion that particular miRNAs are specifically regulated by toxicity-associated signaling events. First, according to published quantitative data of the mouse hippocampal microRNAome [22] TAMs are expressed at low levels in naive hippocampus in vivo. Second, using several stimulation paradigms that are supposed to enhance or mimic synaptic activity, neither we nor others [9, 10, 45] found increased TAM levels. In contrast, several forms of toxicity- or stress-associated signaling like induction of insulin resistance [57], response to diabetic renal injury [58], DNA-damage response [59], and NMDAR induced death signaling (this study) increase the levels of all or a subset of TAMs. The fact that TAMs appear to be exclusively induced by toxicity-associated stimuli raises questions about their biological function. It is conceivable that TAMs are part of an adaptive response to stress and injury. By fine tuning components of stress signaling pathways, TAMs might help to maintain cellular homeostasis under conditions of mild stress. Such a physiological function of TAMs remains to be investigated in future studies, especially given the relatively low expression levels of TAMs compared to other neuronal miRNAs [22]. It would, however, be in line with the emerging view of miRNAs acting as mediators and/or modulators of diverse forms of stress signaling in a variety of cells and tissues [60–63]. Regardless of their biological function, TAMs might be candidate molecules for biomarkers of neurodegeneration [52, 64].

### Implications of TAM co-regulation

Altered miRNA expression has been linked to neural dysfunction before. In some studies, inhibition of a single dysregulated miRNA provided marked effects, like a reduction of neurotoxicity [20], a rescue of age-associated cognitive impairment [22], and protection from seizures [54]. While these studies focused on individual miRNAs with distinct regulatory roles, we here describe an entire set of co-regulated miRNAs. The coordinate change in the levels of multiple TAMs might be required for two reasons. First, individual miRNAs usually have only a modest effect on the expression of their target genes, but different miRNAs can act cooperatively to more strongly regulate their targets [65, 66]. Second, although subtle changes in the expression of individual genes may not have an effect on the phenotype, simultaneously occurring small changes in the levels of several genes can produce biologically meaningful effects [67]. Thus, TAMs could affect excitotoxicity via two mechanisms, i.e., pronounced regulation of a few shared target genes and/or subtle changes in the levels of multiple functionally related targets. In either case, the need for coordinated changes in the expression of several TAMs might serve as a safeguard against spurious activation of a potentially detrimental signaling pathway.

### How does toxic NMDAR-signaling increase TAM levels?

The most obvious mechanism to couple NMDAR activation to increased TAM levels would be the activation of specific transcription factors resulting in increased transcription of specific pri-miRNAs. However, we found the increase in TAM levels to be transcription-independent. By knocking-down Drosha, we found that pri-miRNA processing appears to be required for increases in TAM levels. Technical limitations of these experiments include the incomplete and variable loss of DROSHA protein upon viral expression of an anti-Drosha shRNA. This could perhaps be improved by the use of a different shRNA sequence or by extended duration of shRNA expression to allow for more complete protein turnover. Nevertheless, results from the Drosha knock-down experiments suggest, that changes in TAM levels could be due to a signal-induced change in the expression and/or activity of the miRNA biogenesis machinery. One possible mechanism may involve a Ca^2+^ /calpain-dependent, NMDA-induced cleavage of DICER resulting in the liberation of a DICER fragment with increased processivity [68]. Alternatively, changes in the expression of the pri-miRNA processing enzymes, Drosha and Dgcr8, as have been described in the brains of Huntington’s Disease model mice and after NMDAR stimulation in vitro, could account for increased miRNA levels [10, 69], although such a mode of regulation would not explain why TAMs but not other miRNAs are increased by extrasynaptic NMDAR stimulation. If it is not the regulation by a shared transcription factor, it is conceivable that information encoded in the structure of the pri- or pre-miRNA determines whether or not a miRNA is a TAM. Such a mechanism would be in line with the well-established structure-dependent selective interaction of RNAs with their cognate RNA-binding proteins [70]. In the case of miRNAs, RNA binding proteins such as SRSF1, TRBP and TDP-43 were shown to selectively bind to specific pre-miRNAs via recognition of their terminal loop or stem region to alter their processing rate [71–73].

In summary, our results define a new death signaling-associated pathway that is triggered by the activation of extrasynaptic NMDARs. The regulation of a subset of miRNAs adds to the growing list of processes that are differentially controlled by the survival promoting versus death inducing activities of NMDARs [25, 26].

## Supplementary information

**Additional file 1.** miRNA microArray results.

## Data Availability

Complete microarray data have been deposited in NCBI’s Gene Expression Omnibus and are accessible through GEO Series accession number GSE47601. Other datasets used during the current study are available from the corresponding author on reasonable request.

## Abbreviations

4-AP: 4-aminopyridine
AMPA: α-amino-3-hydroxy-5-methyl-4-isoxazolepropionic acid
AP: action potential
BDNF: brain-derived neurotrophic factor
CaMKII: calcium/calmodulin-dependent protein kinase II
GABA: γ-aminobutyric acid
NMDA: *N*-methyl-D-aspartate
qRT-PCR: quantitative real-time polymerase chain reaction
rAAV: recombinant adeno-associated virus
shRNA: short hairpin RNA
TAM: toxicity-associated microRNA
